# Cultural Adaptation and Evaluation of the Namaste Care Program for Home-Dwelling People With Dementia and Their Caregivers : Protocol for a Mixed Methods Study

**DOI:** 10.2196/78449

**Published:** 2025-11-24

**Authors:** Meng Jin, Dijuan Meng, Chang Sun, Qinan Shen, Yamei Bai, Guihua Xu, Yulei Song, Shanshan Wang, Hongtu Chen

**Affiliations:** 1 School of Nursing, Nanjing University of Chinese Medicine, 138 Xianlin Avenue, Qixia District, Nanjing, Jiangsu, 210023, China, 86 13915947121; 2 School of Nursing, The Hong Kong Polytechnic University, Hong Kong, China; 3 Department of Psychiatry, Harvard Medical School, Boston, MA, United States

**Keywords:** Namaste Care, dementia, caregivers, cultural adaptation, China, home-based care, nonpharmacological interventions, mixed methods

## Abstract

**Background:**

The Namaste Care program is a person-centered intervention that has been shown to improve the quality of life of people with dementia and to alleviate caregiver burden. However, its application in China remains underexplored. Given China’s unique sociocultural norms, cultural adaptation is essential to ensure feasibility, acceptability, and contextual fit.

**Objective:**

The primary objective is to culturally adapt the Namaste Care Home Program to the Chinese community context. The secondary objective is to evaluate the feasibility and acceptability of the adapted program (qualitative analysis), and to explore its preliminary effects (quantitative analysis) on quality of life and related outcomes for people with dementia and their family caregivers.

**Methods:**

A 3-stage, 11-step mixed methods design is used in this study. The process includes a baseline stage for community assessment and stakeholder consultations, a formulation stage for adapting the intervention and training materials, and an execution and evaluation stage in which trained family caregivers deliver the intervention. A 1-group, pretest-posttest design will be used, with quantitative assessments at baseline (T0), immediately after intervention (T1), and at the 3-month follow-up (T2), complemented by qualitative process evaluations.

**Results:**

The study was approved in April 2025 (2025-NZY-4‐01) and registered on April 18, 2025 (ChiCTR2500101042). Recruitment occurred from January to April 2025, enrolling 15 caregiver–people with dementia dyads. As of April 2025, baseline data collection is complete, and analysis is ongoing. Primary results are expected in mid-2026.

**Conclusions:**

The cultural adaptation of the Namaste Care Home Program is a critical step toward advancing person-centered, home-based dementia care in China. Findings will provide evidence on feasibility and cultural appropriateness and will inform the design of future large-scale trials to test effectiveness in cross-cultural settings.

## Introduction

Dementia affects over 55 million individuals globally and is projected to triple by 2050 [1,2], with more than 60% of people with dementia living in low- and middle-income countries (LMICs). China alone accounts for approximately 20% of global cases, posing a major public health challenge [3]. Dementia leads to cognitive decline, behavioral and psychological symptoms of dementia, progressive dependence, and reduced quality of life (QoL) for people with dementia and their families [4-6].

In China, caregiving is predominantly family based under the influence of filial piety, with over 96% of care provided by family members who often lack formal training [7,8]. Consequently, more than 80% of caregivers experience anxiety, depression, and high strain [9,10]. Institutional care exists but is costly and often viewed as temporary, reinforcing reliance on home-based care [11].

Nonpharmacological interventions such as cognitive stimulation therapy [12] and reminiscence therapy [13] are widely used, yet benefits concentrate in the early to middle stages; their reliance on cognitive abilities limits feasibility for advanced dementia and may not align well with China’s family-care context [6,14]. In contrast, Namaste Care requires minimal verbal ability, can be delivered by trained family caregivers at home, is a relatively low resource, and targets comfort and behavioral and psychological symptoms of dementia through multisensory engagement—priorities for moderate-to-advanced dementia in China’s predominantly home-based care [6,12-16].

Developed in Western settings, Namaste Care emphasizes emotional- and sensory-focused engagement and has shown promise in improving QoL among people with moderate-to-advanced dementia [15,16]. However, the existing evidence is primarily from Western contexts, and there is no culturally adapted Namaste Care model that has been evaluated in China [9,17]; thus, the applicability, feasibility, and effectiveness under China’s family-based caregiving norms remain uncertain. Accordingly, this protocol outlines the cultural adaptation of Namaste Care for China and a mixed methods feasibility evaluation to inform subsequent multicenter testing.

The primary objective is to culturally adapt the Namaste Care Home Program (NCHP) for the Chinese community context. The secondary objectives are to: (1) evaluate feasibility (eg, recruitment, retention, and adherence) and acceptability from family caregivers’ perspective (qualitative analysis); (2) explore preliminary effects on QoL among people with dementia (quantitative analysis); and (3) explore preliminary effects on caregivers’ outcomes, including QoL, caregiving burden, caregiving abilities, attitudes toward dementia, and positive aspects of caregiving (quantitative analysis).

## Methods

### Study Design

**Figure 1.**
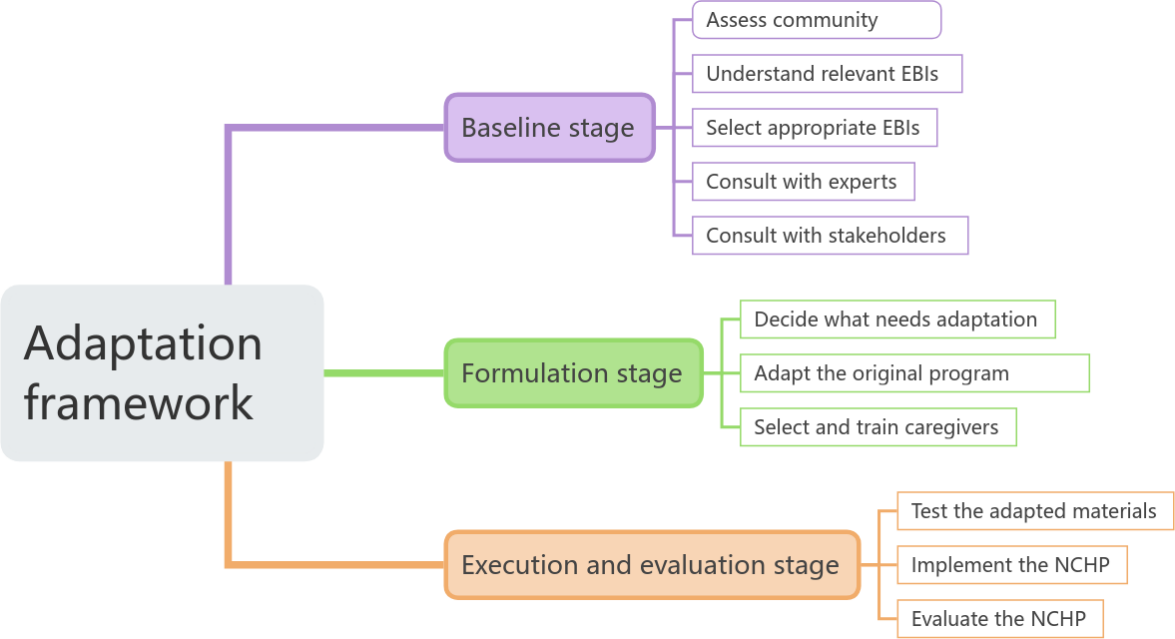
Framework for evidence-based adaptation of interventions. EBIs: evidence-based interventions; NCHP: Namaste Care Home Program.

This protocol uses a 3-stage mixed methods design guided by the 11-step adaptation framework proposed by Escoffery [18]. This structured approach ensures a systematic process, from community needs assessment to intervention implementation and evaluation, as illustrated in Figure 1.

#### Stage 1: Baseline Stage (Steps 1-5)

##### Step 1: Assess Community

**Figure 2.**
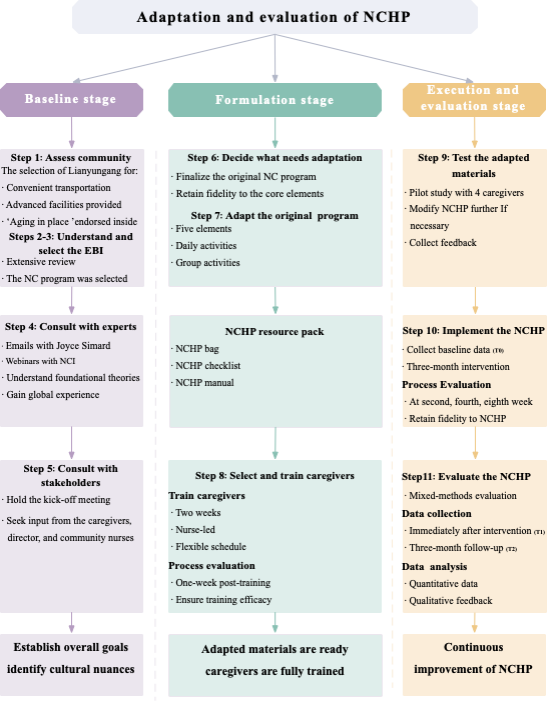
NCHP adaptation and implementation framework. EBI: evidence-based intervention; NCI: Namaste Care International; NCHP: Namaste Care Home Program.

We selected Lianyungang, Jiangsu (Chaoyang Community Health Service Center) based on facility readiness, participant engagement, and transport accessibility, aligned with the local priority of aging in place [19] (ethics approval: 2025-NZY-4‐01; refer to the Ethical Considerations section). The detailed steps are shown in Figure 2.

##### Steps 2-3: Understand and Choose the Evidence-Based Intervention

A comprehensive review of nonpharmacological interventions for moderate-to-advanced dementia informed evidence-based intervention (EBI) selection under resource constraints in LMICs [1]. We selected Namaste Care for its sensory- and emotion-focused components, home deliverability by family caregivers, and stage appropriateness. In addition to these practical considerations, its philosophy, embodied in the respectful *namaste* greeting, aligns with the Chinese cultural values, such as compassionate caregiving and filial piety. A brief comparative rationale versus cognitive stimulation therapy or reminiscence therapy is provided in the Introduction section.

##### Step 4: Consult with Experts

We consulted Joyce Simard (founder of Namaste Care) and Rishi Jawaheer (Honorary Chair, Namaste Care International) through webinars and email exchanges to draw on global adaptation experience and to delineate core components, identify adaptable elements, and gather guidance on community implementation [20].

##### Step 5: Consult with Stakeholders

We conducted structured meetings with caregivers, community directors, and nurses to map care challenges, knowledge gaps, and preferred delivery formats, thereby generating an initial context, mechanism, and outcome specification for adaptation. We anticipate engaging approximately 12 to 18 stakeholders (eg, 8‐12 family caregivers, 2‐3 community directors, and 2‐3 nurses), sampling will continue until information power is adequate for decision-making.

### Stage 2: Formulation Stage (Steps 6-8)

#### Step 6: Decide What Needs Adaptation

The team will identify China-specific modifications while preserving the NCHP’s core objectives, principles, and theoretical foundations. Drawing on initial stakeholder feedback and a review of implementations in other regions, we will specify changes to setting, materials, and training so that activities are practical for home use and can be integrated into daily routines. Community nurses, caregivers, and family members will be convened to finalize these adaptations, ensuring alignment with Chinese caregiving values and practice norms. Outputs will include a China-adapted specification that distinguishes core from adaptable elements.

#### Step 7: Adapt the Original Program

We will complete the conceptual translation and simplify and standardize procedures to support delivery by community nurses and family caregivers. Daily activities will be tailored to the capabilities of people with dementia, and brief group sessions will be scheduled twice weekly to encourage regular participation (Textbox 1).

Textbox 1. **Adapted Namaste Care Home Program (NCHP) delivery schedule (daily routines and twice-weekly group activities).**

**Daily activities**

**Morning sessions**

- Preparatory work

-  Food and drink preparation: seasonal availability, individual preference, swallowing condition
-  Personal care items: NCHP bags (eg, hand cream, face cream)
-  Room setup: soft lighting, soothing aromatherapy, relaxing music

- Life care and activities

-  Morning greetings
-  Facial care: facial cleansing, applying face cream, and rubbing face
-  Hand care: hand cleaning, hand cream massage
-  Traditional Chinese medicine health care: swallowing exercises, finger exercises, meridian tapping, head soothing, finger combing hair, tooth tapping

- Nutritional supplements

-  Snacks: lotus root powder, sesame paste
-  Intermittent hydration: water, Chinese floral teas, soy milk

**Noon sessions**

- Abdominal massage (Fairy kneading manipulation)
- Nap for 20‐30 minutes

**Afternoon sessions**

- Activities

-  Creative activities: paper cutting, painting, colorful doodling, reminiscence therapy, doll therapy
-  Games: tossing game, card game
-  Traditional Chinese cultural activities: listening to opera, crosstalk, and storytelling
-  Outdoor activities: low-angle sunbathing (3‐5 PM) and walking

- Nutritional supplements

-  Food: fruit platter or fruit puree
-  Intermittent hydration: water, milk (avoid drinks that affect sleep)

**Evening sessions**

- Life care
  - Foot care: warm water foot soak, foot massage
  - Sleep care: Chinese herbal aromatherapy for sleep aid (Acorus calamus, Albizia Flower, etc), applying body lotion

- Finish work

-  Touching the back of the hand and patting the back
-  Praising the people with dementia for their cooperation throughout the day
-  Document the people with dementia’s activity preferences and complete the NCHP checklist

**Group activities (twice weekly)**

- Competitive activities: marble grabbing and ring toss game
- Cultural and social gatherings: Red Song parties and tea parties
- Creative activities: horticultural therapy, flower arrangement, DIY handicrafts, and creating a family calendar
- Festival Activities: making rice dumplings and sachets, making mooncakes, paper cutting, and making dumplings

**Figure 3.**
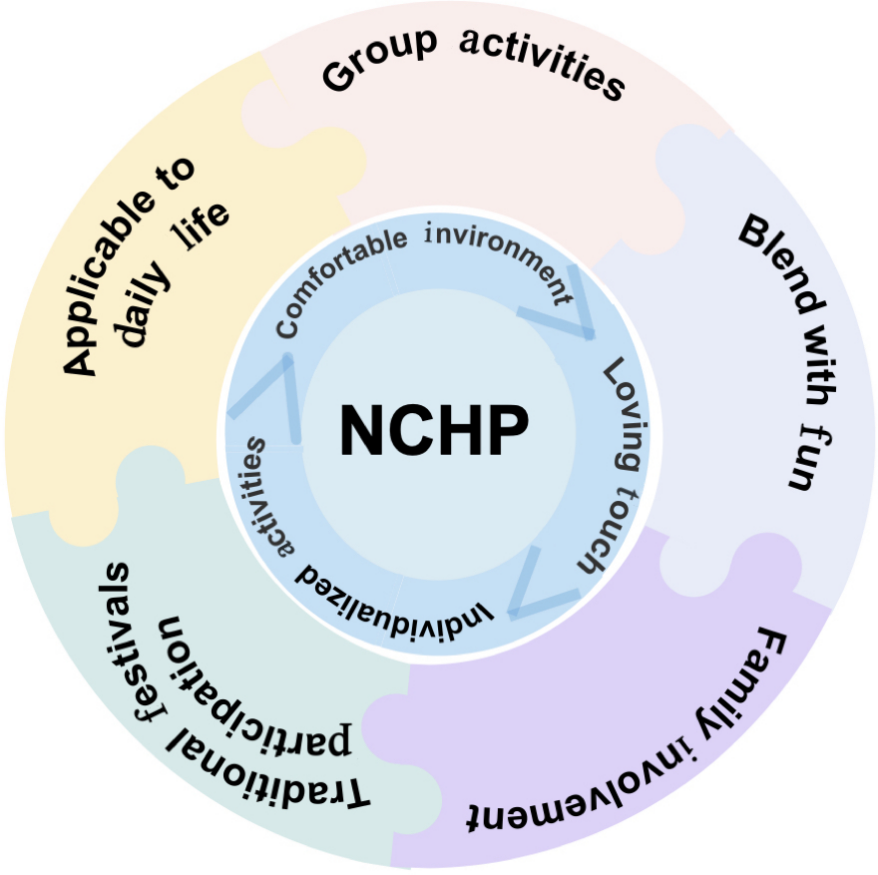
Five elements of the adapted Namaste Care Home Program (NCHP) are depicted here.

The adapted package will retain the program’s core principles while addressing 5 elements (Figure 3): (1) applicability to daily life (affordable, familiar items, eg, Pechoin SOD emulsion; Tong Ren Tang vitamin E lotion and selected traditional Chinese medicine–informed practices such as head soothing and finger combing; food choices that meet nutritional and swallowing needs); (2) group activities that foster social and cultural engagement (eg, paper cutting, Red-Song gatherings, and tea parties); (3) blending with fun through enjoyable and functional games (eg, tossing and card games) to stimulate cognition and mobility; (4) family involvement via tasks that promote participation and strengthen bonds (eg, family calendars and joint activities); and (5) traditional festival participation to increase relevance and feasibility.

The adapted materials will be reviewed by Joyce Simard to ensure fidelity to the core principles. To assess acceptability and practicality, we will conduct 2 to 3 focus group (6‐8 participants each) discussions with community nurses, caregivers, and family members [20], guided by the Bowen feasibility framework and acceptability theory [21,22]; interview guides (Table S1 in Multimedia Appendix 1) will be used to structure data collection [23]. Additionally, 10 to 15 semistructured caregiver interviews will be conducted, and data collection will continue until adequate information power and evidence of theme saturation are reached. Semistructured interviews will also elicit feedback on the activity checklist and item list to finalize personalized NCHP checklists and NCHP bags, and caregivers will review the NCHP manual.

#### Step 8: Select and Train Caregivers

We will use criterion sampling [21] to recruit eligible dyads. Eligibility criteria for people with dementia include (1) age 60 years or older; (2) moderate-to-severe dementia as determined by the Mini-Mental State Examination [22], clinical symptoms, and caregiver report, confirmed by a trained evaluator; (3) medically stable; and (4) community-dwelling (noninstitutionalized). Caregiver eligibility criteria include (1) age 18 years or older; (2) provision of continuous, 24-hour in-home care for at least 3 months; and (3) ability to communicate effectively.

**Figure 4.**
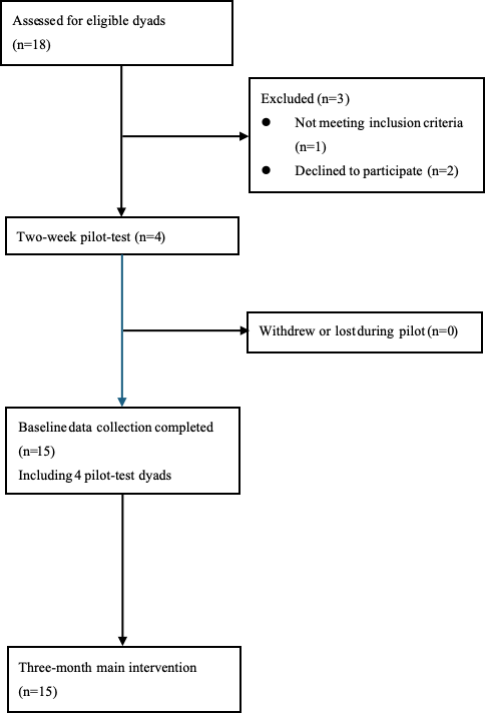
CONSORT-style participant flow for the 1-group feasibility protocol.

We will recruit a feasibility sample of 10 to 15 dyads to assess recruitment, retention, adherence, and acceptability rather than to test hypotheses [23]. Previous feasibility studies of Namaste Care and similar interventions have used comparable sample sizes [24], and Bowen et al [25] recommend using small sample sizes for exploratory feasibility objectives. Eighteen dyads were screened, and 3 were excluded, 4 dyads completed a 2-week pilot test, and all 15 dyads then proceeded to the 3-month intervention. (See Figure 4 for participant flow).

### Pilot Sample-Size Justification

This feasibility study prioritizes estimation precision over hypothesis testing. Therefore, we adopt a precision-based rationale for 10 to 15 dyads. For feasibility proportions (eg, 3-month retention and weekly adherence), a sample of 15 dyads yields an approximate 95% CI half-width of about +22 or –22 percentage points when the true rate is around 75% (and about +27 or –27 percentage points for n=10), which is sufficient to judge performance against feasibility thresholds. For continuous outcomes, a sample of 10 to 15 dyads provides approximately 24% to 19% relative precision for the SD (RSE(σ)≈√[1/(2(n−1))]), which is adequate to inform sample-size calculations for a subsequent trial. Regarding safety, if no adverse events (AEs) are observed, the “rule of three” places the upper 95% confidence bound on per-dyad AE risk at approximately 3/n (ie, no more than 20% for n=15), providing an acceptable early-phase safety screen. Taken together and consistent with previous feasibility studies of Namaste Care and related dementia interventions, a sample of 10 to 15 dyads is sufficient and appropriate to estimate key feasibility metrics and guide the design of a larger study.

### Training Plan

Training will be led by the principal investigator (a Namaste Care regional champion) with the support of community nurses [26]. Sessions will be delivered on weekends across 2 consecutive weeks, approximately 2 hours per day, supplemented by online modules to enhance access and optional individualized follow-ups for emerging challenges. The curriculum will cover the program’s history and core practices; practical use of the NCHP checklist and NCHP bag; assessment of pain, mood, and agitation; and illustrative activities (eg, memory books and dementia-friendly gardening). A concise NCHP manual will be provided before training and will be used as an ongoing reference. Following training, caregivers will apply skills at home with ongoing support, and a process evaluation 1 week after training will assess engagement and inform iterative updates to the manual and training [26].

### Stage 3: Execution and Evaluation Stage (Steps 9‐11)

#### Step 9: Test the Adapted Materials

We will recruit 4 people with dementia–caregiver dyads for a 2-week pilot to test the adapted NCHP in home settings [27]. After providing written informed consent, caregivers will implement the NCHP at home and use a daily checklist to record activities, task completion, and any tailoring (including adjustments to the NCHP bag as needs evolve). Upon completion, the principal investigator will conduct a focus group discussion with caregivers to collect feedback on feasibility, acceptability, and procedural clarity [28]; field notes will inform refinements prior to full implementation.

#### Step 10: Implement the NCHP

The adapted NCHP will then be implemented over 3 months in Lianyungang, with the research team providing ongoing supervision and responsive adjustments as needed. Baseline (T0) data will be collected using structured questionnaires before the intervention. For people with dementia, outcomes will include QoL and self-perceived burden; for caregivers, outcomes will include QoL, caregiver burden, caregiving capacity, attitudes toward dementia, and positive caregiving feelings. The purpose, instruments, and assessment schedule for both groups are specified in Tables1  2.

**Table 1.** Data collection for people with dementia.

Data collected and tool used	Primary purpose	Time points of data collection		
		Baseline (T0)	Immediately after intervention (T1)	3-month follow-up (T2)
Sociodemographics	Age, gender, ethnicity, medical conditions, and access to social support such as family, friends, or formal care resources	✓	—^a^	—
QOL-AD^b^	Assess quality of life of people with dementia	✓	✓	✓
SPBS^c^	Assess the self-perceived burden of people with dementia	✓	✓	✓

a Not applicable.

b QOL-AD: Quality of Life-Alzheimer Disease scale; increased score reflects improved quality of life; the total score ranges from 13 to 52.

c SPBS: Self-Perceived Burden Scale; decreased scores reflect less perceived burden; total score ranges from 10 to 50.

**Table 2.** Data collection time points for caregivers.

Data collected and tool used	Primary purpose	Time points of data collection		
		Baseline (T0)	Immediately after intervention (T1)	3-month follow-up (T2)
Sociodemographics	Age, gender, educational level, family structure (eg, relationship to the patient and presence of children or other dependents), living situation (eg, cohabiting with the patient, living alone, or in a care facility), and caregiving experience (eg, duration of caregiving)	✓	—^a^	—
WHOQOL-BREF^b^	Assess quality of life of caregivers	✓	✓	✓
C-ZBI^c^	Assess caregiver burden	✓	✓	✓
CTI-25^d^	Assess the caregiving capacity	✓	✓	✓
FAS-C^e^	Assess attitudes toward dementia	✓	✓	✓
C-PAC^f^	Assess positive feelings toward caregiving	✓	✓	✓

a Not applicable.

b WHOQOL-BREF: World Health Organization Quality of Life Scale- Brief Form Questionnaire; increased scores reflect improved quality of life; total score ranges from 0 to 100.

c C-ZBI: Zarit Burden Interview, Chinese version; decreased scores reflect less caregiver burden; total score ranges from 0 to 88.

d CTI-25: Caregiver Task Inventory-25; decreased scores reflect less complexity of caregiving tasks; total score ranges from 0 to 50.

e FAS-C: Family Attitude Scale, Chinese version; decreased scores reflect less adverse relational effects; total score ranges from 0 to 150.

f C-PAC: Positive Aspects of Caregiving Scale; increased scores reflect more positive caregiving emotions; the total score ranges from 0 to 55.

The NCHP coordinator will conduct regular check-ins with caregivers; during the first 2 weeks, reviews will be intensified to identify scheduling or checklist issues and enact immediate adjustments. Process evaluations will be conducted at weeks 2, 4, and 8 [29]. All protocol deviations (eg, withdrawal of consent and discontinuation) will be documented.

#### Step 11: Evaluate the NCHP

We will use a mixed methods approach that integrates quantitative and qualitative data to assess the feasibility, acceptability, and preliminary effects.

### Process 1: Data Collection

This feasibility study will use a 1-group pretest-posttest design. We acknowledge potential threats to internal validity (eg, history and regression to the mean), noting that this design is standard in feasibility research preceding more rigorous trials.

#### Feasibility Metrics

Feasibility benchmarks will be based on prior work [30] and will include the following: (1) NCHP application frequency (goal: ≥3 days per week, with ≥4 hours on at least 1 of those days); (2) retention over 3 months (goal: ≥75%, consistent with dementia-care feasibility studies [31]); and (3) incidence of AEs (goal: 0%; potential AEs include skin breakdown and falls). Although we aim for 0%, any AE will be closely monitored and addressed per protocol [32]. These thresholds will guide go, modify, or stop decisions for a subsequent trial: ≥75% for both retention and adherence=proceed; 60%‐74% for both=proceed with modifications; <60% for either metric=redesign, conditional on safety (no serious adverse events [SAEs] and all AEs appropriately managed).

#### Qualitative Acceptability

Postintervention (T1) interviews will explore acceptability and caregiver experience, including perceived advantages and disadvantages, satisfaction with implementation, barriers and facilitators, and suggestions for improvement [33]. The interview guides are provided in Table S2 in Multimedia Appendix 2.

#### Quantitative Outcomes

Quantitative assessments will be conducted at baseline (T0), immediately after intervention (T1), and 3-month follow-up (T2). The primary outcome will be QoL, assessed using the Quality of Life-Alzheimer Disease scale for people with dementia (caregiver-report form; Cronbach α=0.87 [34]) and the World Health Organization Quality of Life Scale-Brief Form Questionnaire (WHOQOL-BREF) for caregivers (validated Chinese version; Cronbach α=0.70‐0.88 [35]). Secondary outcomes will include the Self-Perceived Burden Scale (Cronbach α=0.91 [36]) for people with dementia and the Positive Aspects of Caregiving Scale, Chinese version (Cronbach α=0.89 [37]), the Caregiver Task Inventory-25 (Cronbach α=0.93 [38]), the Zarit Burden Interview, Chinese version (Cronbach α=0.87 [39]), and the Family Attitude Scale, Chinese version (Cronbach α=0.93 [40]) for caregivers.

All measures have published psychometric support in Chinese samples; detailed properties and assessment schedules are shown in Tables1  2. Where available, instruments show acceptable test–retest reliability and sensitivity to change; where evidence is limited, we will prioritize feasibility signals and effect-size estimation to guide outcome selection for the subsequent trial.

### Process 2: Data Analysis

#### Quantitative Analysis

We will summarize demographics with descriptive statistics and analyze repeated measures using linear mixed-effects models at T0, T1, and T2 under a missing-at-random assumption for incomplete follow-up. Given the small pilot and feasibility focus, models will be parsimonious (time as a categorical fixed effect; random intercept only), estimated by restricted maximum likelihood with Satterthwaite small-sample degrees-of-freedom corrections in SPSS software (version 26; IBM Corp). We will report marginal means and effect sizes with 95% CIs, with primary emphasis on precision estimates; any *P* values will be descriptive. Diagnostics (eg, residuals) will be reviewed, with simple transformations or robust SEs applied if needed. Sensitivity analyses will use generalized estimating equations with small-sample-corrected robust SEs and complete-case paired summaries. We will also report variance components (random-intercept variance and intraclass correlation) to inform the design of subsequent, adequately powered trials.

#### Qualitative Analysis

With written consent, interviews will be audio-recorded and analyzed using Braun and Clarke reflexive thematic analysis [41]. To enhance trustworthiness, we will use investigator triangulation (2 independent coders, consensus meetings, and third-reviewer adjudication), member checking with a subset of participants (conducted after preliminary theme maps to verify clarity and interpretability rather than enforce agreement), and an audit trail comprising decision logs, codebook versioning, theme maps, analytic memos, and meeting minutes. Coding will be managed in NVivo (version 14; Lumivero), with analysis proceeding concurrently with data collection. A codebook will be developed iteratively (initially seeded from focus-group responses); discrepancies will be resolved by consensus rather than interrater coefficients, consistent with reflexive thematic analysis. We will document researcher reflexivity through brief positionality statements and reflexive journals. Sampling sufficiency and stopping will be guided primarily by information power, with evidence of theme saturation used to corroborate stopping decisions. Codes will be organized into themes and subthemes, and a final member check will confirm the interpretations.

#### Mixed Methods Integration

Quantitative and qualitative findings will be triangulated to generate meta-inferences about feasibility, acceptability, and contextual fit, thereby informing final refinements to the NCHP and the design of subsequent larger-scale studies [41,42].

### Ethical Considerations

The study was approved by the Nanjing University of Chinese Medicine Ethics Committee in April 2025 (2025-NZY-4‐01) and was registered at the Chinese Clinical Trial Registry on April 18, 2025 (ChiCTR2500101042). The study protocol adhered to the principles of the Declaration of Helsinki [43] and Good Clinical Practice guidelines. All participants provided written informed consent before participation.

Given the nature of the population involved (people with dementia), special consideration was given to obtaining informed consent. Although dementia leads to cognitive decline, it does not automatically imply a loss of decision-making capacity. People with dementia may retain the ability to understand simplified consent forms and express their preferences, especially in the early stages. The study respected the residual autonomy of participants, and information was adapted to accommodate their cognitive abilities. Consent was continuously monitored throughout the study. In cases where a participant could not provide valid consent, a legally designated representative was involved, but the participant’s willingness to participate was always considered through a “double consent” approach. Participants had the right to withdraw at any time without any consequences. As compensation for their time and effort, each participating dyad received a 100 RMB (US $14.07) gift package upon completion of assessment. This was clarified during the consent process to avoid undue influence.

AEs include skin breakdown, falls, or activity-related discomfort, defined as any unfavorable and unintended sign, symptom, or event occurring during participation. SAEs are defined as events that result in death, life-threatening conditions, hospitalization or its prolongation, significant disability, or other medically significant conditions. Caregivers will be trained to recognize and document AEs and to report them immediately to the NCHP coordinator. The research team will assess severity and relatedness, determine whether an event is intervention-related, and take appropriate action. SAEs will be reported to the principal investigator and the ethics committee within 24 hours; nonserious AEs will be documented within 72 hours and summarized in periodic safety reports. Immediate first aid will be provided when necessary, and participants will be referred to community health services if required. If repeated or severe intervention-related AEs occur, the intervention may be paused, or the participant withdrawn; study-level suspension will be considered if predefined safety thresholds are exceeded. An independent safety officer will conduct an independent safety review at regular intervals to oversee AE logs and corrective actions.

Personal data collected during the trial will be handled and stored in accordance with applicable data protection laws, including the General Data Protection Regulation and the Personal Information Protection Law of China. Data will be pseudonymized upon study completion, and access to identifiable data will be restricted to authorized study personnel. All data and related documentation will be securely stored in compliance with regulatory requirements, and the use of study data will be controlled by the principal investigator.

## Results

Recruitment was conducted from January to April 2025 and yielded 15 caregiver–people with dementia dyads (n=30). Caregivers had a mean age of 65.7 (SD 11.7) years; 60% (9/15) were women, 66.7% (10/15) were married, and 73.3% (11/15) were retired or not working. People with dementia had a mean age of 83.5 (SD 6.8) years; 60% (9/15) were men, 66.7% (10/15) were in the moderate stage of dementia, 33.3% (5/15) were in the late stage, and 46.7% (7/15) were married, while 53.3% (8/15) were widowed. Recruitment is now closed, baseline assessments are complete, and data analysis is ongoing. Detailed participant characteristics are presented in Tables3  4.

**Table 3.** Characteristics of caregivers (n=15).

Characteristics	Participants, n (%)	
Age (years)		
45‐59	4 (26.7)	
60‐74	6 (40)	
≥75	5 (33.3)	
Gender		
Men	6 (40)	
Women	9 (60)	
Nonbinary	0 (0)	
Marital status		
Married	10 (66.7)	
Widowed	2 (13.3)	
Divorced	3 (20)	
Employment status		
Working	4 (26.7)	
Not working or retired	11 (73.3)	

**Table 4.** Characteristics of people with dementia (n=15).

Characteristics	Participants, n (%)	
Age (years)		
75‐79	4 (26.7)	
80‐84	6 (40)	
≥85	5 (33.3)	
Gender		
Men	9 (60)	
Women	6 (40)	
Nonbinary	0 (0)	
Marital status		
Married	7 (46.7)	
Widowed	8 (53.3)	
Stage of dementia category		
Moderate	10 (66.7)	
Late	5 (33.3)	

## Discussion

### Anticipated Findings

This protocol describes the systematic cultural adaptation and feasibility evaluation of the NCHP for Chinese families. By tailoring this EBI to local cultural values and practical realities, we aim to deliver a feasible and acceptable program that enhances QoL for people with dementia and reduces caregiver burden.

The use of a structured adaptation framework provides a transparent, replicable roadmap for modifying EBIs to new contexts and may inform similar work in other LMICs. Integrating culturally relevant elements (eg, traditional Chinese medicine–informed practices and locally meaningful activities) is expected to support engagement and acceptability. The feedback-driven, iterative process is designed to keep the intervention person-centered while maintaining fidelity to the core components.

Findings from this feasibility study will inform the design of future larger studies. Feasibility estimates (recruitment yield, 3-month retention, session adherence, and variance of primary outcomes) will inform sample-size calculations and site-level targets; fidelity and workload data will help determine training dose, supervision cadence, and acceptable fidelity thresholds; and process metrics (eg, time on task, missed visits, and data-capture completeness) will guide whether to standardize phone-based or brief digital assessments at scale.

Although this study is expected to provide valuable insights, several limitations must be considered. First, the one-group pretest-posttest design limits our ability to draw causal inferences, as observed changes may reflect history, regression to the mean, or the Hawthorne effect. Second, the small sample size and single-site design will constrain generalizability. Additionally, measurement bias is possible (eg, caregiver-reported outcomes), and blinding will not be feasible. To mitigate these issues, we will prespecify outcomes and assessment time points (T0, T1, and T2), monitor fidelity and AEs, integrate qualitative data for triangulation, and place primary emphasis on effect sizes with 95% CIs rather than hypothesis testing. Subsequent studies will adopt comparative designs (eg, waitlist, cluster randomized, or stepped-wedge trials) to strengthen causal inference and external validity.

### Anticipated Challenges and Methodological Contingencies

We anticipate risks related to caregiver adherence, participant attrition, and resource constraints (eg, time, staffing, and materials), as well as potential intervention contamination within community settings. To address these risks, we plan to (1) provide scheduled check-ins and just-in-time coaching to sustain adherence; (2) use practical supports (brief manuals and NCHP bags) and flexible scheduling to reduce burden; (3) monitor fidelity with brief checklists and remedial feedback; (4) prospectively document protocol deviations and reasons for missing data; and (5) apply analysis plans suitable for small feasibility samples (eg, parsimonious linear mixed-effects models under a missing-at-random assumption, descriptive *P* values, and sensitivity analyses). If adherence or retention falls below predefined feasibility thresholds (eg, ≥3 days per week, with ≥4 hours on ≥1 day, and ≥75% retention), we will implement prespecified adjustments, such as increasing remote support frequency, simplifying activity menus, or modifying data-collection modes (eg, phone-based or brief digital questionnaires), while documenting all changes in the audit trail. These contingencies will inform modifications for a subsequent adequately powered multisite evaluation.

### Conclusions

The findings of this study will be crucial for informing future research. Specifically, data on feasibility (recruitment and retention rates), acceptability (caregiver feedback), and preliminary effect sizes will provide foundational insights for the design of larger-scale, multicenter, randomized controlled trials. Such a trial would offer definitive evidence of the effectiveness and cost-effectiveness of the adapted NCHP, paving the way for its broader dissemination and integration into the Chinese community-based long-term care system.

This culturally adapted NCHP holds significant potential in addressing the unique challenges faced by Chinese family caregivers of people with dementia, offering a holistic, person-centered intervention that aligns with local caregiving practices. The outcomes of this study will contribute valuable knowledge on the feasibility and acceptability of the program, setting the stage for future studies to confirm its effects. These efforts will ultimately strengthen the evidence base for nonpharmacological dementia interventions in China and other resource-limited settings.

## Supplementary material

10.2196/78449 Multimedia Appendix 1 Personal interview guide and focus group guide for the formulation stage.

10.2196/78449 Multimedia Appendix 2 Personal interview guide for the execution and evaluation stage.

10.2196/78449 Checklist 1 SPIRIT checklist.

## Abbreviations

EBI: evidence-based intervention
LMIC: low- and middle-income country
NCHP: Namaste Care Home Program
QoL: quality of life
WHOQOL-BREF: World Health Organization Quality of Life Scale-Brief Form Questionnaire
