# Supplementary material for: Cultural Adaptation and Evaluation of the Namaste Care Program for Home-Dwelling People With Dementia and Their Caregivers : Protocol for a Mixed Methods Study
Source: JMIR Res Protoc. 2025 Nov 24;14:e78449. doi: 10.2196/78449 (PMC12643391; doi:10.2196/78449)
Supplement: Multimedia Appendix 1 [file resprot-v14-e78449-s001.docx]

**Supplementary files for online publication only**

**Table S1.** Personal interview guide and focus group guide for the formulation stage

| **Formulation stage** |
| --- |
| **Focus Group Interview Guide** |
| 1. **Acceptability:**  - What are your impressions of the initial draft of this adapted NCHP^1^? - Do you believe it will be accepted by caregivers of home-dwelling Chinese older adults with moderate to severe dementia? Why or why not? - What factors might deter individuals from participating?  1. **Demand:**  - How significant do you believe the demand for this adapted NCHP is among caregivers of PWD^2^ in China? - What unmet needs do caregivers face when taking care of these home-dwelling PWD? - Can this intervention address these unmet needs? - What specific needs can it meet, and which ones might it be unable to meet?  1. **Implementation:**  - What do you believe are the biggest challenges in implementing this adapted NCHP? - Are the resources we currently provide sufficient to facilitate the implementation of this intervention? - What additional resources or conditions are needed to help implement this intervention? - How can we ensure the smooth implementation of this intervention?  1. **Practicality:**  - How practical can caregivers apply this intervention in their daily caregiving routines? - How much time and effort might be required? - What potential obstacles could they encounter?  1. **Adaptation:**  - What adjustments are needed to make this intervention more suitable for local conditions? - What specific modifications do you suggest? - What cultural or social factors should be mainly considered?  1. **Expansion:**  - What potential do you see for expanding this intervention within our province and other regions of China? - What additional support and resources would be required to implement this intervention on a larger scale? - What key factors could promote successful expansion?  1. **Impacts:**  - We have initially designed this intervention plan. What do you think are the potential effects of this intervention? - What specific outcomes or changes would you expect to see? - What indicators could be used to evaluate its effectiveness?  1. **Integration:**  - How can this intervention be integrated into your daily caregiving routine? - What challenges might arise during the integration process? - What support do you think is needed to ensure smooth integration? |
| **Personal Interview Guide** |
| **NCHP checklist:**  1. How do you perceive PWD’s acceptance of the checklist? Are there any items particularly well-received? Why?  2. Are any checklist components that must be better adapted to your caregiving situation? If so, what specific changes and support would be necessary to adjust these components better to suit your needs?  **NCHP bag:**   1. What materials would you like in the NCHP bag (e.g., scents, lotions, soaps, balls, etc.)? Are there any culturally specific items that should be considered? 2. How appropriate are you to find the adapted NCHP bag items? 3. Can you share specific examples of how the NCHP bag has assisted or could assist in your caregiving activities? What improvements would make it more helpful?   **NCHP manual:**   1. What are your thoughts on the training manual? How well does the NCHP manual align with the scheduled activities outlined in the program? 2. Does the manual adequately address cultural considerations relevant to your caregiving context? 3. Does the manual provide adequate guidance on overcoming common challenges faced during the implementation of the NCHP? 4. What additional support or examples could be included to assist caregivers better? |
| **Progress Evaluation** |
| **Training:**   1. What are your general thoughts on the NCHP training program? 2. What aspects of the NCHP training did you find most challenging or difficult to grasp? 3. How confident do you feel in implementing the NCHP after attending the training? 4. Did the training provide clear and actionable guidance on how to use the items in the NCHP checklist and NCHP bag? |

^[[1]](#footnote-0)^NCHP: Namaste Care Home Program

^[[2]](#footnote-1)^PWD: people with dementia

Note: The guide was inspired by the Bowen feasibility and acceptability framework (Bowen, 2009)

1. [↑](#footnote-ref-0)
2. [↑](#footnote-ref-1)
