# Supplementary material for: Cultural Adaptation and Evaluation of the Namaste Care Program for Home-Dwelling People With Dementia and Their Caregivers : Protocol for a Mixed Methods Study
Source: JMIR Res Protoc. 2025 Nov 24;14:e78449. doi: 10.2196/78449 (PMC12643391; doi:10.2196/78449)
Supplement: Multimedia Appendix 2 [file resprot-v14-e78449-s002.docx]

**Supplementary files for online publication only**

**Table S2.** Personal interview guide for the execution and evaluation stage

| **Execution and evaluation stage** |
| --- |
| **Personal Interview Guide** |
| **Experience of NCHP^1^:**   1. Could you share your success stories from implementing the NCHP at home? 2. How has the NCHP affected your overall caregiving experience? 3. How confident and comfortable did you feel in implementing the NCHP at home? 4. Will you find it easier to provide localized activities? 5. How have you adapted the NCHP activities to fit better the local context and the specific needs of the PWD^2^ you care for? 6. What adaptations have you made to cater to their preferences and conditions? 7. How well do you think the NCHP has been adapted to fit the cultural context of your community? 8. What aspects of the NCHP have worked well for you? Can you describe a moment that you feel best reflects the effect of participation in the program on PWD? 9. Can you describe a situation during the NCHP implementation that you remember being very positive? 10. What challenges have you encountered while implementing the NCHP? How have you addressed these challenges, and what solutions have you found effective?   **Effects of NCHP:**   1. What impact did the NCHP have on you? 2. What impact did the NCHP have on PWD? 3. Has the NCHP changed your perspective as a caregiver? If so, what are the differences? 4. How has the NCHP affected your relationship with PWD? 5. To what extent do you think the training has helped you to implement the NCHP? 6. What is your opinion of the NCHP checklist, NCHP bag, and information material we provided to you? Do you have any suggestions for us? 7. Would you suggest any specific cultural training or resources to help implement the NCHP more effectively? 8. Which checklist components do not align well with your caregiving situation and require adaptation? Could you specify the necessary changes and support to facilitate these adaptations?   **Satisfaction with NCHP：**   1. Does the NCHP match your expectations before starting? 2. How satisfied were you in implementing the program? 3. Do you plan to continue using the NCHP in caregiving? Why or why not? 4. Would you recommend the NCHP to other PWD caregivers? Why or why not? 5. What, in your opinion, is essential to continue successfully offering the NCHP to PWD who live at home in the future? 6. Based on your experience, what cultural considerations would you recommend be included in future iterations of the NCHP to enhance its effectiveness and acceptance in your community? |

^[[1]](#footnote-0)^NCHP: Namaste Care Home Program

^[[2]](#footnote-1)^PWD: people with dementia

Note: The guide was inspired by the Bowen feasibility and acceptability framework (Bowen, 2009)

1. [↑](#footnote-ref-0)
2. [↑](#footnote-ref-1)
